# *Tenebrio molitor* larvae meal inclusion affects hepatic proteome and apoptosis and/or autophagy of three farmed fish species

**DOI:** 10.1038/s41598-021-03306-8

**Published:** 2022-01-07

**Authors:** Eleni Mente, Thomas Bousdras, Konstantinos Feidantsis, Nikolas Panteli, Maria Mastoraki, Konstantinos Ar. Kormas, Stavros Chatzifotis, Giovanni Piccolo, Laura Gasco, Francesco Gai, Samuel A. M. Martin, Efthimia Antonopoulou

**Affiliations:** 1 School of Veterinary Medicine, Laboratory of Ichthyology- Culture and Pathology of Aquatic Animals, Aristotle University of Thessaloniki, GR-54124 Thessaloniki, Greece; 2 Department of Ichthyology and Aquatic Environment, School of Agricultural Sciences, University of Thessaly, Fytoko street, GR38446 Volos, Greece; 3 Laboratory of Animal Physiology, Department of Zoology, School of Biology, Aristotle University of Thessaloniki, 541 24 Thessaloníki, Greece; 4 Institute of Marine Biology, Biotechnology and Aquaculture, Hellenic Centre for Marine Research, Gournes Pediados, P.O. Box 2214, 71003 Heraklion, Crete, Greece; 5 Department of Veterinary Medicine and Animal Production, University of Naples Federico II, Via F. Delpino 1, 80137 Naples, Italy; 6 Department of Agricultural, Forest and Food Sciences, University of Turin, Largo P. Braccini 2, 10095 Grugliasco, Italy; 7 Institute of Sciences of Food Production, National Research Council, Largo P. Braccini 2, 10095 Grugliasco, Italy; 8 School of Biological Sciences, University of Aberdeen, Tillydrone Avenue, Aberdeen, UK

**Keywords:** Zoology, Ichthyology

## Abstract

Herein, the effect of dietary inclusion of insect (*Tenebrio molitor*) meal on hepatic pathways of apoptosis and autophagy in three farmed fish species, gilthead seabream (*Sparus aurata*), European seabass (*Dicentrarchus labrax*) and rainbow trout (*Oncorhynchus mykiss*), fed diets at 25%, 50% and 60% insect meal inclusion levels respectively, was investigated. Hepatic proteome was examined by liver protein profiles from the three fish species, obtained by two-dimensional gel electrophoresis. Although cellular stress was evident in the three teleost species following insect meal, inclusion by *T. molitor*, *D. labrax* and *O. mykiss* suppressed apoptosis through induction of hepatic autophagy, while in *S. aurata* both cellular procedures were activated. Protein abundance showed that a total of 30, 81 and 74 spots were altered significantly in seabream, European seabass and rainbow trout, respectively. Insect meal inclusion resulted in individual protein abundance changes, with less number of proteins altered in gilthead seabream compared to European seabass and rainbow trout. This is the first study demonstrating that insect meal in fish diets is causing changes in liver protein abundances. However, a species-specific response both in the above mentioned bioindicators, indicates the need to strategically manage fish meal replacement in fish diets per species.

## Introduction

Sustainable aquaculture production has never been more crucial due to the expanding export and the increased consumption of seafood products. To this end, development and improvement of aquaculture sustainable practices are in great need of research and knowledge concerning fish nutrition. Finding alternative feed ingredients and protein sources for the aqua feed industry due to insufficient global supplies of fishmeal (FM)^1,2^ is an active area in fish nutrition research.

Insect larvae meals are considered a very promising alternative to provide valuable proteins for aqua feeds and have been in the spotlight of many researches^3–6^. The European Commission approved the use of processed animal protein from insects in feeds for aquaculture (Reg. EU 2017/893) and their use is expected to dramatically increase^7,8^. Different insect species are considered for the production of larvae meal and among them yellow mealworm (*Tenebrio molitor*—TM) is one of the most promising. Recent studies reported positive results in different fish species^9–13^ as well as crustaceans^14^ even though, high levels of inclusion could lead to a slight decrease of performances, especially in fish juveniles^9,15^.

Diet-induced oxidation stress leading to immune dysfunction has been previously reported in many studies^16–18^. Oxidative stress can activate two different closely linked cell mechanisms, autophagy and apoptosis^19^. Autophagy, a lysosome-mediated mechanism pivotal in the regulation of cellular damage, targets dysfunctional proteins and cytoplasmic organelles for degradation and recycling^19,20^, in order to avoid apoptosis. However, autophagy and apoptosis can coexist since both can be triggered by similar stressors which activate the upstream signaling pathway^21^. If apoptosis, which is a mitochondria-related pathway of programmed cell death in multicellular organisms, is triggered, the activation of caspases cleaves for the damaged cells^22^. Therefore, key components of the aforementioned cell pathways, that are widely characterized according to the existing literature as indicators of physiological and dietary stress, may elucidate if nutrient changes in aquafeeds maintain a welfare regime. Such stress indicators include Bax and Bcl-2 proteins, which promotes and prevents apoptosis respectively, caspases which finally induce apoptosis and are in turn activated when the relative amount of Bax is higher than Bcl-2, and LC3 II/I ratio and SQSTM1/p62 which are basic constituents of the autophagosomes and indicators of the autophagic process.

Moreover, to determine mechanisms of diet-host response, methods and techniques (genomics, transcriptomics, proteomics, metabolomics and bioinformatics) are applied to evaluate the new feed ingredients in order to ensure fish health and performance^23^. In proteomics analysis, the abundance of many proteins is measured simultaneously and can provide indications of the affected metabolic pathways. Proteomic studies have improved the understanding of the relationships between diet composition, protein metabolism and nutrient utilisation in aquatic animals^24–26^. Proteomics approaches have been used extensively to examine protein responses to dietary stimulations in both liver and muscle in rainbow trout (*Oncorhynchus mykiss*)^26–29^, zebrafish^30,31^, Atlantic salmon^32^, gilthead seabream and white sea bream^25,26,33^. This paper investigated whether the dietary inclusion of *T. molitor* meal affected hepatic pathways of apoptosis and autophagy in three farmed fish species, gilthead seabream (*Sparus aurata*), European seabass (*Dicentrarchus labrax*) and rainbow trout (*Oncorhynchus mykiss*). Due to the fact that changes in mRNA and protein levels can strongly correlate or not (e.g., transcriptional and translational control regulation) (e.g.,^34–36^), and although the examination of differences between transcriptional and translational events is of great interest, this study mainly focused on the biochemical responses as a result of translational processes, in order to be consistent to the proteome analysis presented herein. Thus, this study investigated the effect of insect meal inclusion on the liver proteome of the aforementioned species to assess the magnitude of changes occurring in hepatic proteins expression and to give indications of how the different species may adapt to the diet modification.

## Material and methods

### Dietary experiments and sampling

Three independent dietary trials were conducted using three farmed fish species; gilthead seabream, European seabass and rainbow trout. The experiments are described in detail for European seabass in Gasco et al.^9^ and for gilthead seabream in Piccolo et al.^11^. As partial fish meal substitution, the same full-fat TM larvae meal, purchased from the Gaobeidian Shannong Biology CO. LTD (Shannong, China) (Italy) (DGSFA 0019960-P) (02/11/2012), was used in the fish diets. Ingredients and proximate composition of experimental diets are reported in Table 1^9,11^. Formulated diets were designed to meet the different nutritional requirements of each fish species.

**Table 1:** Composition of the experimental diets.

Composition (g/kg)	*S. aurata*		*D. labrax*		*O. mykiss*	
	0%	25%	0%	50%	0%	60%
Fish meal	500	330	700	200	700	100
Wheat gluten	–	–	50	150	–	–
Wheat flour	–	–	92	80	40	40
Wheat bran	–	–	55	25	57	50
Corn gluten	150	125	0	0	0	37
Starch (gelatinized, D500)	180	170	0	12	33	100
*T. molitor* meal	0	250	0	500	0	600
Fishoil	140	95	90	20	150	53
Trace metals supplement	10	10	–	–	10	10
Vitamins supplement	10	10	–	–	10	10
Binder	10	10	–	–	–	–
Methionine	–	–	6	6	–	–
Lysine	–	–	3	3	–	–
Choline	–	–	1.5	1.5	–	–
Vitamins and trace metals mixture (Premix)	–	–	2.5	2.5	–	–
Total	1000	1000	1000	1000	1000	1000

Gasco et al.^9^ and Piccolo et al.^11^.

The experimental protocols were designed according to the guidelines of the current European Directive (2010/63/EU) on the protection of animals used for scientific purposes. The gilthead seabream trial was performed at the Department of Veterinary Medicine and Animal Production (University of Naples Federico II, Italy), as described in Piccolo et al.^11^, and was approved by the Ethic Committee of Federico II University. The European seabass trial was performed at the Institute of Marine Biology, Biotechnology and Aquaculture (IMBBC) of the Hellenic Center for Marine Research (Crete, Greece) (EL91- BIOexp-04), as described in Gasco et al.^9^ and approved by the Aquaexcel Ethic Committee (Ref 0013/03/05/15B and Ref. 0125/08/05/15/TNA). The rainbow trout experiment was approved by the Ethic Committee and was conducted at the registered experimental facility of the DISAFA (Torino, Italy) (DM n. 182/2010) by accredited scientists. All methods are reported in accordance with ARRIVE guidelines.

Briefly, as described in Piccolo et al.^11^, gilthead seabream juveniles of 105.2 ± 0.17 g average initial body weight were fed two isoenergetic and isoproteic diets, for 163 days. These were a control diet (TM0) in which fish meal (FM) was the main protein source and TM25 diet in which 25% of TM larvae meal was added to the diet as partial substitution of FM. European seabass juveniles (initial body weight 5.2 ± 0.82 g) were fed two isonitrogenous, isolipidic and isoenergetic diets where TM was included at a level of 0% or 50% (well above the recommended level of 10%) as partial substitution of FM (further details in Gasco et al.^9^) and the feeding trial lasted 70 days. Finally, a 90-day trial was conducted on rainbow trout (initial body weight:115.2 ± 14.2 g), which was fed two experimental diets having 0% or 60% (well above the recommended level of 10%) of TM inclusion, as described in Antonopoulou et al.^37^. In all trials, fish were fed to apparent satiation, gilthead seabream and European seabass were fed 7 days per week whereas the rainbow trout was fed 6 days per week.

At the end of each growth trial, 10 healthy fish from each dietary group were removed and sacrificed by aneasthesia overdose (tricaine methanesulfonate-MS222, Sigma Aldrich, St. Louis, MO, USA), 24 h following their final meal. The fish body weight was measured and the liver was sampled and kept in -80 °C for proteomic analysis.

### Protein extraction and gel analysis

Proteins from liver of gilthead seabream, European seabass and rainbow trout were identified by 2DE gels. Protein extraction and analysis were performed in line with Cash et al.^38^ and Martin et al.^28^. As described in Mente et al.^26^ liver samples were kept cool and using a pestle were homogenized in 2-D lysis buffer [0.5 ml 0.5 M Tris–HCl pH 6.8, 0.125 ml 0.2 M EDTA, 12 g urea (8 M), 2.5 ml 0.5 M DTT, 2.5 ml glycerol (10%), 1.25 ml NP-40 (5%), 3.7 ml pH 3–10 ampholytes (40%) 6%, 5 ml MilliQ water]. Lysis buffer was added in a 10:1 ratio and the homogenates were centrifuged at 11,000× g for 10 min. The supernatants were collected and stored at − 80 °C. Proteins (in the supernatants) were precipitated by using a ReadyPrep 2-D Clean up kit (Bio-Rad Laboratories, Hercules USA) following the manufacturer’s instructions. The precipitate was solubilized in 200 µL IPG buffer [(2.01 g UREA (7 M), 0.76 g Thiourea (2 M), 0.2 g CHAPS (4%), 0.015 g DTT (0.3%), 3 ml MilliQ water, 50 μl pH 4–7 IPG buffer (GE Healthcare)] and in sufficient bromophenol blue to provide to the solution a blue color. The protein solution was sonicated with 3 bursts each of 5 s and then was incubated with one part of DNase solution (0.05 ml 1 M MgCl_2_, 0.5 ml 1 M Tris–HCl pH 8.0 and 0.1 ml 20,000 U ml/1) to two parts protein solution for 10 min on ice. The protein samples were analyzed by 1-dimensional SDS PAGE to check protein quality and concentration prior to 2 DE. Following isoelectric focusing, IPG strips were applied to the second dimension SDS-PAGE (Criterion AnykD Gel, Bio-Rad), electrophoresed and the resolved proteins detected using Colloidal Coomassie Blue G250 staining. The gels were dried and scanned in an Image ScannerTMIII (GE Healthcare, UK) with LabScan software (GE Healthcare, UK). 16 bit images were obtained in a resolution of 600 dpi. The digitilised images were transferred to the Progenesis SameSpots, version 4.5 (Non-linear Dynamics, Newcastle upon Tyne, UK). A reference gel from the control samples was selected.

Comparison of the 2D protein profiles between fish fed the 0% TM inclusion dietary treatment and the 25%, 50% and 60% TM inclusion, was carried out using 4 biological replicates. The 2D protein profiles for each fish and treatment were matched to the 2D reference gel within Progenesis SameSpots software. Protein spots showing statistically significant differences in abundance between the three species groups per treatment were selected using ANOVA (*p* < 0.05). Each species was analyzed independently as it is not possible to compare across species as 2 DE (2-dimension gel electrophoresis) proteome patterns are highly different between species. A reference gel was chosen for each species to represent the “proteome map”.

### SDS/PAGE, ubiquitin and cleaved caspases conjugates, and immunoblot analysis

The preparation of tissue samples for SDS-PAGE, quantification of caspases and ubiquitinated proteins and the immunoblot analysis are based on well-established protocols. Specifically, for the SDS-PAGE in the present study, equivalent amounts of proteins (50 μg), from livers from 5 individual animals from each species and diet regime, were separated either on 10% and 0.275% or 15% and 0.33% (w/v) acrylamide and bisacrylamide respectively. Thereafter, they were electrophoretically transferred onto nitrocellulose membranes. Antibodies used were as follows: monoclonal rabbit anti-LC3B (3868, Cell Signaling), polyclonal rabbit anti-p62/SQSTM1 (5114, Cell Signaling), anti-Bcl2 (7973, Abcam) and anti-Bax (B-9) (2772, Cell Signaling). Quantification of caspases and ubiquitinated proteins was assessed in a solid- phase immunochemical assay. The antibodies used were a polyclonal anti-ubiquitin rabbit antibody (Cat. No. 3936, Cell Signalling, Beverly, MA, USA) and anti-cleaved caspase antibody (Cat. No.8698 Cell Signalling, Beverly, MA, USA).

### Statistics

Changes in apoptosis and autophagy indicators were tested for significance at the 5% level by using one way ANOVA [GraphPad Instat 3.10 (GraphPad Instat Software)]. Post-hoc comparisons were performed using the Bonferroni test. Values are presented as means ± S.D.

### Ethical statement

The experimental protocols were designed according to the guidelines of the current European Directive (2010/63/EU) on the protection of animals used for scientific purposes. The gilthead sea bream trial was performed at the Department of Veterinary Medicine and Animal Production (University of Naples Federico II, Italy), as described in Piccolo et al. (2017) and was approved by the Ethic Committee of Federico II University. The European seabass trial was performed at the Institute of Marine Biology, Biotechnology and Aquaculture (IMBBC) of the Hellenic Center for Marine Research (Crete, Greece) (EL91- BIOexp-04), as described in Gasco et al. (2016) and approved by the Aquaexcel Ethic Committee (Ref 0013/03/05/15B and Ref. 0125/08/05/15/TNA). The trout experiment was approved by the Ethic Committee and conducted at the registered experimental facility of the DISAFA (DM n. 182/2010) by accredited scientists.

## Results

### Protein profile

**Figure 1:**
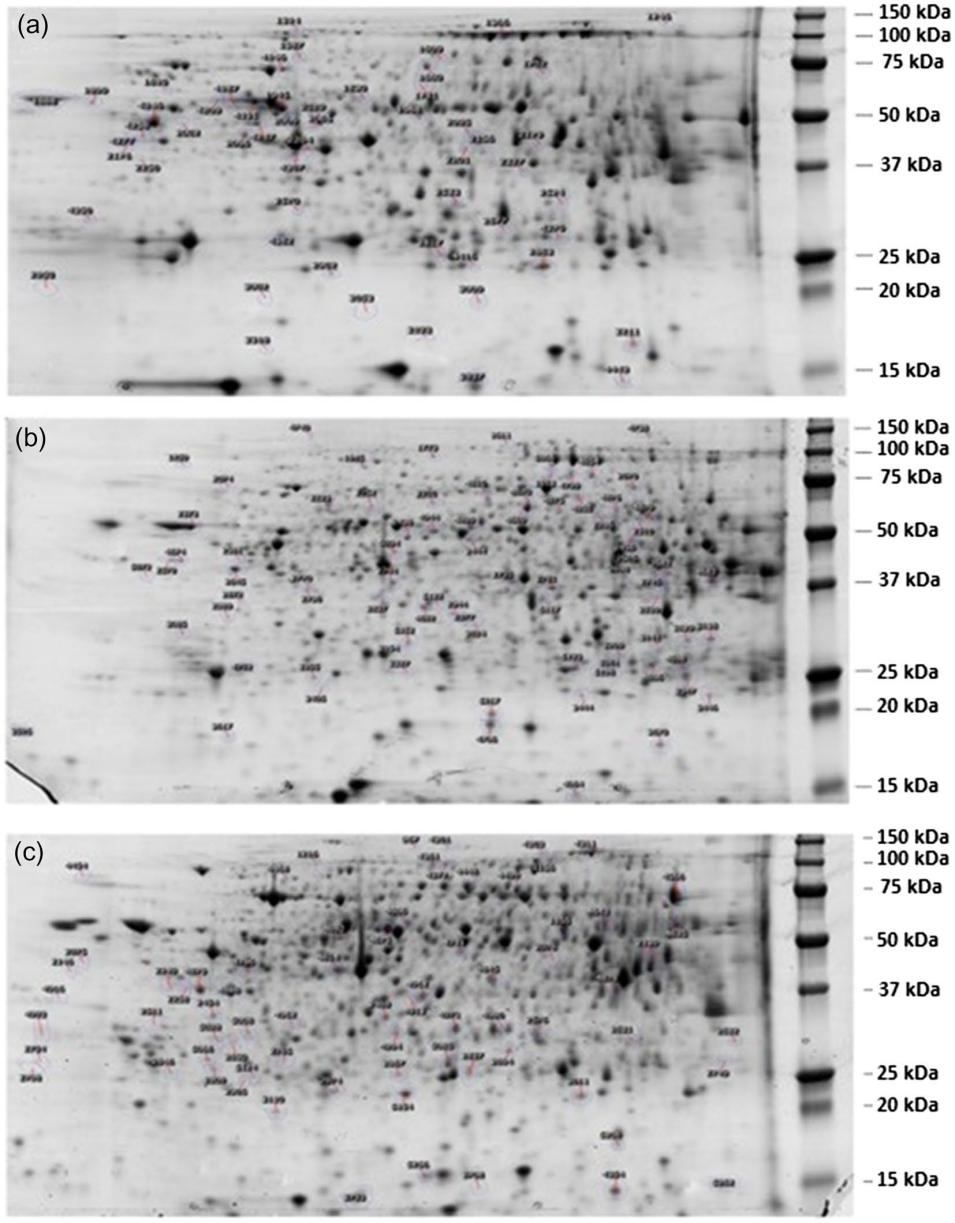
A representative two-dimensional gel of liver proteins of (**a**) gilthead seabream (*Sparus aurata*), (**b**) European seabass (*Dicentrarchus labrax*) and (**c**) rainbow trout (*Oncorhynchus mykiss*) fed 0% inclusion of *T. molitor* (TM) meal. Protein spots showing significant changes in their abundance are indicated by a number.

The protein profile from a fish at the 0% insect (TM) meal inclusion shows a representative sample of the liver proteins separated by 2DE and it represents the reference gel for gilthead seabream, European seabass and rainbow trout (Fig. 1). The gels show high resolution of the cellular proteins with *pI* of 4–7 and molecular weights of 10–150 kDa.

**Figure 2:**
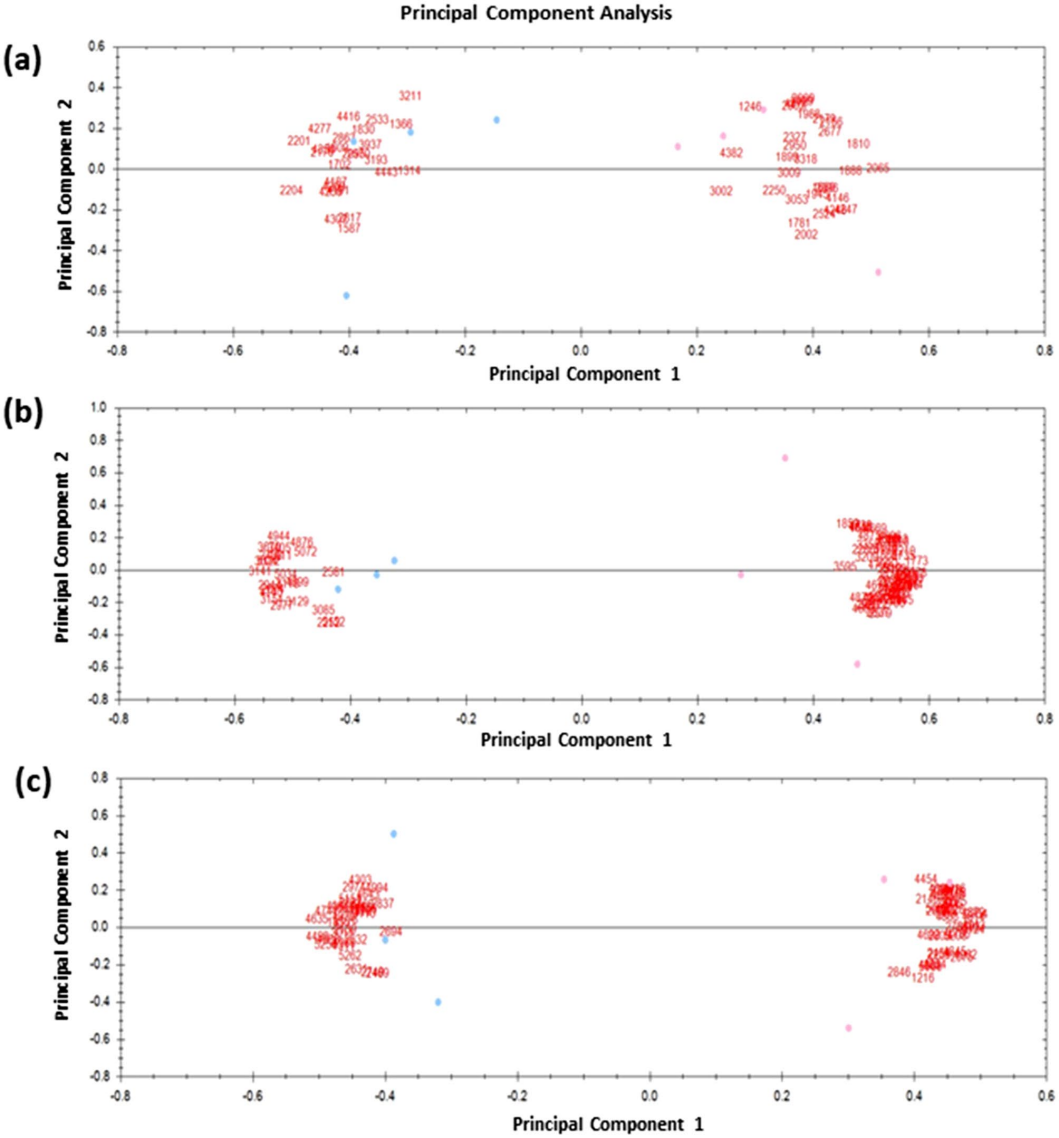
PCA changes in abundance of proteins spots. The 2D gel images were grouped per dietary inclusion of *T. molitor* (TM) meal for (**a**) gilthead seabream (*Sparus aurata*), (**b**) European seabass (*Dicentrarchus labrax*) and (**c**) rainbow trout (*Oncorhynchus mykiss*). The locations of the significantly different protein spots (expressed as mean normalised values) [Progenesis SameSpots version 4.5 (Non-linear Dynamics, Newcastle upon Tyne, UK www.nonlinear.com)] from four or five gel images per dietary treatment were used.

The number of protein spots identified across all gels varied from 500 to 900. Following quality control and editing, 550 spots for each species were obtained for statistical analysis in all gels, among which 30, 81 and 72 spots were found to differ significantly (ANOVA, *p* < 0.05) in abundance in gilthead seabream, European seabass and rainbow trout, respectively (Table 2). We found fourteen, twenty-three and thirty-three spots that increased in abundance, at the 25%, 50% and 60% insect (TM) meal inclusion in gilthead seabream, European seabass and rainbow trout, respectively. The magnitude of the protein abundances for significant spots in the three dietary groups ranged between 1.24 and 2.25 for gilthead seabream, 1.17 to 3.2 for European seabass and 1.8–3.3 for rainbow trout. Sixteen, fifty-eight and forty proteins were found decreased in abundance in gilthead seabream, European seabass and rainbow trout, respectively. The mean normalized protein spot volume for the 60% insect (TM) meal inclusion was expressed significantly different in abundance than the 0% insect (TM) meal inclusion group in rainbow trout (*p* < 0.05). There were no significant differences in abundance of the mean normalized protein spot volume at the dietary treatments of the 25 and 50% insect (TM) meal inclusion (*p* > 0.05). To further assess the dietary impact on the proteome, PCA analysis characterized the spots according to their abundance levels amongst the three species in relation to their TM inclusion dietary treatment (Fig. 2). According to the PCA, 58% spots were significantly more abundant in proteins in gilthead sea bream in the 25% fishmeal replacement by TM meal cluster, compared to the abundance of 79% spots in the European seabass at the 50% insect (TM) meal inclusion and the 79% spots at the 60% insect (TM) meal inclusion in rainbow trout.

**Table 2:** Protein spots affected upregulated and downregulated by species (mean values for 4 to 5 determinations).

Spots no upregulated*	Anova (*p*)	Fold difference	Species	Spots no downregulated**	Anova (*p*)	Fold	Species
1314	0.04	1.32	sea bream	4246	0.05	1.24	sea bream
1366	0.03	1.55	sea bream	4247	0.05	1.74	sea bream
1587	0.05	1.92	sea bream	4370	0.02	1.36	sea bream
1609	0.04	1.88	sea bream	1810	0.01	1.7	sea bream
1702	0.05	1.47	sea bream	1888	0.03	1.56	sea bream
2095	0.02	1.74	sea bream	1988	0.04	1.79	sea bream
2176	0.05	1.62	sea bream	2009	0.05	1.81	sea bream
2201	0.01	1.43	sea bream	2029	0.04	1.45	sea bream
2204	0.01	1.74	sea bream	2065	0.01	1.53	sea bream
2533	0.02	1.55	sea bream	2096	0.03	1.28	sea bream
2817	0.02	1.38	sea bream	2156	0.06	1.17	sea bream
3193	0.03	1.82	sea bream	2179	0.01	1.43	sea bream
4277	0.04	1.79	sea bream	2327	0.04	1.58	sea bream
4350	0.02	1.71	sea bream	2677	0.03	1.38	sea bream
				2902	0.02	1.59	sea bream
				2950	0.04	2.23	sea bream
1611	0.04	2.26	seabass	1773	0	2.29	seabass
2074	0.03	1.68	seabass	1845	0.04	1.74	seabass
2252	0.04	1.48	seabass	1859	0.04	1.53	seabass
2438	0.01	1.52	seabass	1866	0.04	1.9	seabass
2581	0.05	1.39	seabass	1913	0.01	2	seabass
2944	0.02	1.65	seabass	2070	0.02	2.26	seabass
2977	0.02	1.91	seabass	2210	0	1.97	seabass
3024	0.03	1.21	seabass	2221	0.01	2.05	seabass
3085	0.03	1.42	seabass	2286	0.04	2.33	seabass
3118	0.03	1.72	seabass	2310	0.02	2.78	seabass
3129	0.02	1.43	seabass	2373	0	2.36	seabass
3141	0.01	3.85	seabass	2444	0	1.44	seabass
3154	0.02	1.88	seabass	2456	0.02	1.58	seabass
3405	0.02	1.3	seabass	2505	0	1.37	seabass
3670	0.03	2.41	seabass	2579	0.04	2.52	seabass
4740	0.01	1.82	seabass	2645	0.02	2.16	seabass
4876	0.03	1.34	seabass	2682	0.01	2.74	seabass
4899	0.03	1.78	seabass	2698	0.02	1.87	seabass
4944	0.05	1.97	seabass	2735	0.01	2.43	seabass
5034	0.01	1.96	seabass	2744	0.03	1.78	seabass
5072	0.03	1.35	seabass	2745	0.01	2.26	seabass
5122	0.02	2.47	seabass	2770	0.02	2.41	seabass
5152	0.05	1.53	seabass	2781	0.04	1.43	seabass
1156	0.02	1.84	trout	2790	0.02	1.73	seabass
1855	0.02	2.24	trout	2837	0.03	2.23	seabass
2139	0.02	1.45	trout	2839	0.04	2.21	seabass
2469	0.05	1.23	trout	2872	0.02	1.74	seabass
2576	0.02	1.59	trout	2989	0.04	1.49	seabass
2631	0.02	1.76	trout	3209	0.05	1.72	seabass
2632	0.04	2.36	trout	3261	0.02	2.11	seabass
2694	0.04	1.35	trout	3327	0.01	1.38	seabass
2749	0.05	1.92	trout	3347	0.01	2.22	seabass
2837	0.04	1.25	trout	3355	0.04	1.25	seabass
2867	0.03	1.36	trout	3444	0.02	2.08	seabass
2974	0.01	1.67	trout	3446	0.05	2.12	seabass
3011	0.03	1.29	trout	3595	0.04	1.97	seabass
3110	0.03	1.32	trout	3617	0.04	1.52	seabass
4303	0.03	1.64	trout	4064	0.03	1.58	seabass
4311	0.02	3.19	trout	4654	0.04	1.87	seabass
4361	0.02	1.67	trout	4669	0.01	3.06	seabass
4372	0.03	2.16	trout	4670	0.03	2.55	seabass
4448	0.01	2.23	trout	4674	0.04	2.19	seabass
4468	0.02	1.39	trout	4683	0.03	1.92	seabass
4499	0	1.74	trout	4692	0.01	2.28	seabass
4566	0	1.71	trout	4696	0.02	1.67	seabass
4635	0	1.94	trout	4698	0	1.61	seabass
4643	0.02	1.29	trout	4702	0.02	2.08	seabass
4741	0	2.52	trout	4706	0.02	3.89	seabass
4878	0.03	1.87	trout	4738	0.03	3.18	seabass
4971	0.01	1.43	trout	4799	0.01	2.22	seabass
4980	0.03	1.39	trout	4810	0.02	2.31	seabass
4994	0.04	1.38	trout	4850	0.03	1.31	seabass
5085	0.01	2.13	trout	4875	0.04	1.58	seabass
5154	0.02	2.09	trout	4879	0.04	1.41	seabass
5256	0	1.4	trout	5117	0	2.39	seabass
5262	0.02	2.96	trout	5193	0.03	2.4	seabass
				5198	0.03	1.84	seabass
				5217	0.02	3.46	seabass
				967	0.01	1.54	trout
				1216	0.04	2.72	trout
				2075	0	2.03	trout
				2078	0.02	1.94	trout
				2146	0.03	1.43	trout
				2249	0.02	1.38	trout
				2258	0.03	1.73	trout
				2454	0.04	1.2	Trout
				2650	0.03	1.5	trout
				2754	0	2.32	trout
				2765	0.03	1.6	trout
				2798	0.02	2.68	trout
				2809	0.05	1.37	trout
				2846	0.05	1.65	trout
				2905	0.02	1.45	trout
				3708	0.02	2.36	trout
				3793	0	1.96	trout
				4301	0.04	1.42	trout
				4354	0.03	2.23	trout
				4454	0.04	1.52	trout
				4606	0.03	1.28	trout
				4675	0.03	1.24	trout
				4692	0.05	1.58	trout
				4786	0.01	1.42	trout
				4814	0.01	1.44	trout
				4845	0.01	1.3	trout
				4879	0	1.75	trout
				4888	0.01	2.01	trout
				4912	0.03	1.22	trout
				4913	0	2.24	trout
				4916	0.02	1.81	trout
				4962	0.01	1.76	trout
				4993	0.04	2.04	trout
				5008	0.03	1.65	trout
				5028	0.01	1.47	trout
				5066	0.03	1.73	trout
				5124	0.01	1.84	trout
				5208	0.02	1.58	trout

*Significant difference (upregulated) at 25% TM inclusion compared to 0% TM inclusion (*p* < 0.05) for gilthead seabream, at 50% TM inclusion compared to 0% TM inclusion (*p* < 0.05) for European seabass and at 60% TM inclusion compared to 0% TM inclusion (*p* < 0.05) for rainbow trout (t-test, *p* < 0.05).

**Significant difference (downregulated) at 25% TM inclusion compared to 0% TM inclusion (*p* < 0.05) for gilthead seabream, at 50% TM inclusion compared to 0% TM inclusion (*p* < 0.05) for European seabass and at 60% TM inclusion compared to 0% TM inclusion (*p* < 0.05) for rainbow trout.

### Apoptosis

**Figure 3:**
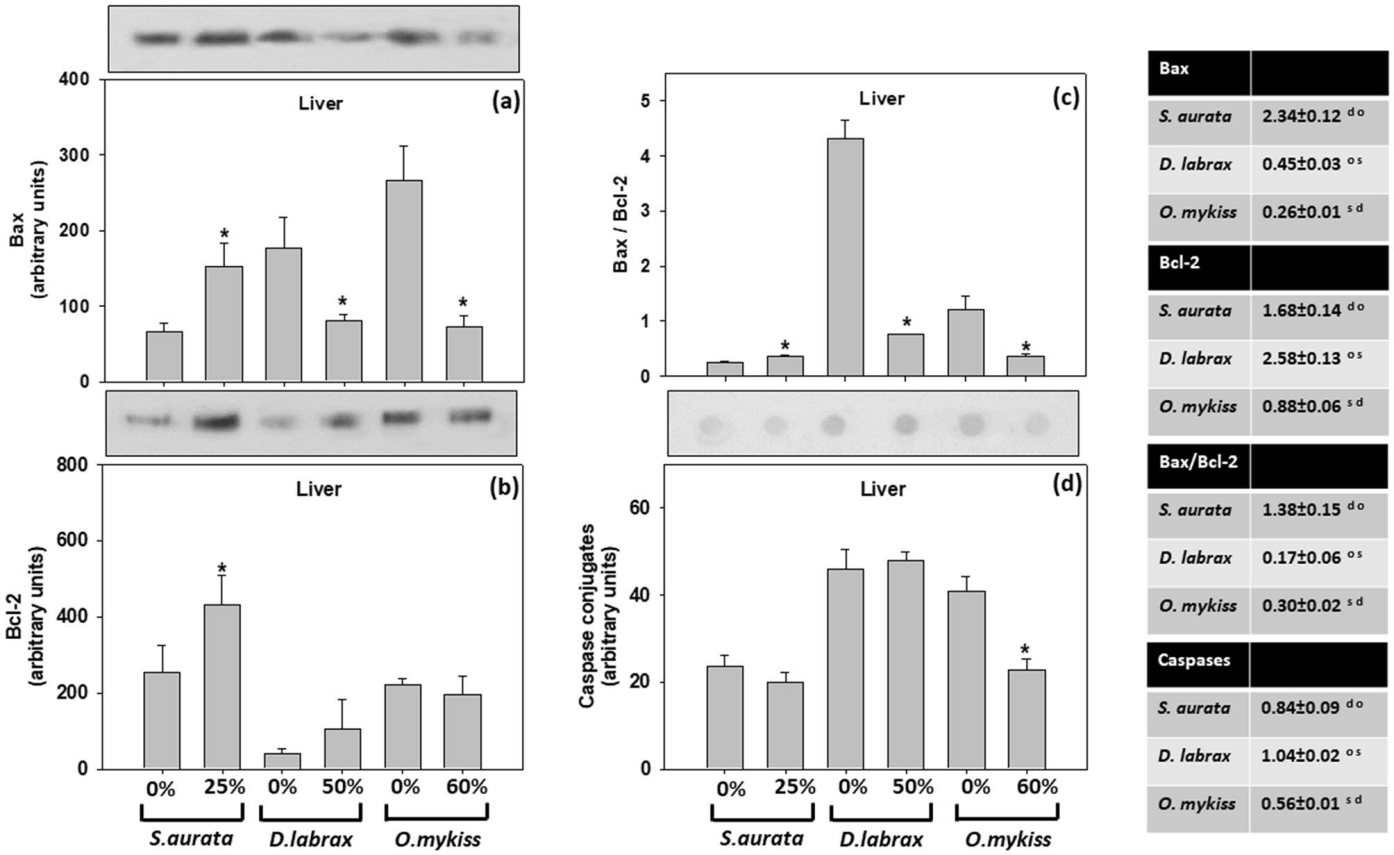
**(a**) Bax, (**b**) Bcl-2, (**c**) Bax/Bcl-2 ratio and (**d**) caspases in the liver of gilthead seabream (*Sparus aurata*), European seabass (*Dicentrarchus labrax*) and rainbow trout (*Oncorhynchus mykiss*) under the 0–25%, 0–50% and 0–60% respectively inclusion of *T. molitor* (TM) meal. Representative immunoblots (Western blot for Bax and Bcl-2; Dot blot for caspases) are shown and were quantified by laser scanning [Gel-Pro Analyzer 4.0 software (Media Cybernetics, Inc. www.mediacy.com)] and plotted [SigmaPlot 12.5 software (Systat Software Inc. www.systatsoftware.com)]. Values represent means ± SD; n = 5 preparations from different animals. Data was statistically analyzed [GraphPad Instat 3.10 (GraphPad Software, www.graphpad.com)]: *denotes significant differences (*p* < 0.05) compared with 0%. The accompanying tables exhibit fold-differences between TM diet inclusion and control (0%). s, d and o denote significant differences (*p* < 0.05) between *S. aurata, D. labrax and O. mykiss,* respectively.

In the sea bream, feeding with insect meal at 25% insect (TM) meal inclusion resulted in a significant increase (*p* < 0.05) of Bax in the liver compared to the control (0%). In the European seabass, a significant reduction (*p* < 0.05) of Bax was observed in the liver with the 50% fishmeal replacement by TM meal. In the rainbow trout, Bax significantly decreased (*p* < 0.05) in the liver of 60% insect (TM) meal inclusion (Fig. 3a).

In the sea bream, significantly increased (*p* < 0.05) expression of Bcl-2 was observed in the liver at 25% insect (TM) meal inclusion. In the European seabass, no significant differences were observed in the liver between 0 and 50% diet treatment (*p* > 0.05). Similarly, in the rainbow trout, no differences were observed in the liver of 0% and 60% insect (TM) meal inclusion treated fish (*p* > 0.05) (Fig. 3b).

Pro-apoptotic and pro-survival proteins (Bax/Bcl-2) ratio finally results in pro-caspases and subsequently caspase activation. After their activation, caspases are substantially involved in the pro-apoptotic pathway which finally induces apoptosis^39,40^. Concerning Bax/Bcl-2 ratio, while in the sea bream, the 25% insect (TM) meal inclusion significantly increased levels of this ratio, in the European seabass and rainbow trout, the 50% and 60% diet treatments respectively, significantly reduced Bax/Bcl-2 ratio (*p* < 0.05) (Fig. 3c).

In the gilthead sea bream and European seabass, no significant differences were observed regarding caspase-conjugated proteins in the liver of 0–25% and 0–50% insect (TM) meal inclusion treated fish, respectively. In the rainbow trout the levels of caspase-conjugated proteins were significantly reduced (*p* < 0.05) in the 60% insect (TM) meal inclusion fish group (Fig. 3d).

Regarding interspecies differences (accompanying tables in Fig. 3), the TM inclusion showed statistically significant differences (expressed as x fold differences compared to control—0%) in Bax, Bcl-2, caspases levels, and Bax/Bcl-2 ratio between all three examined species.

### Ubiquitination and autophagy

**Figure 4:**
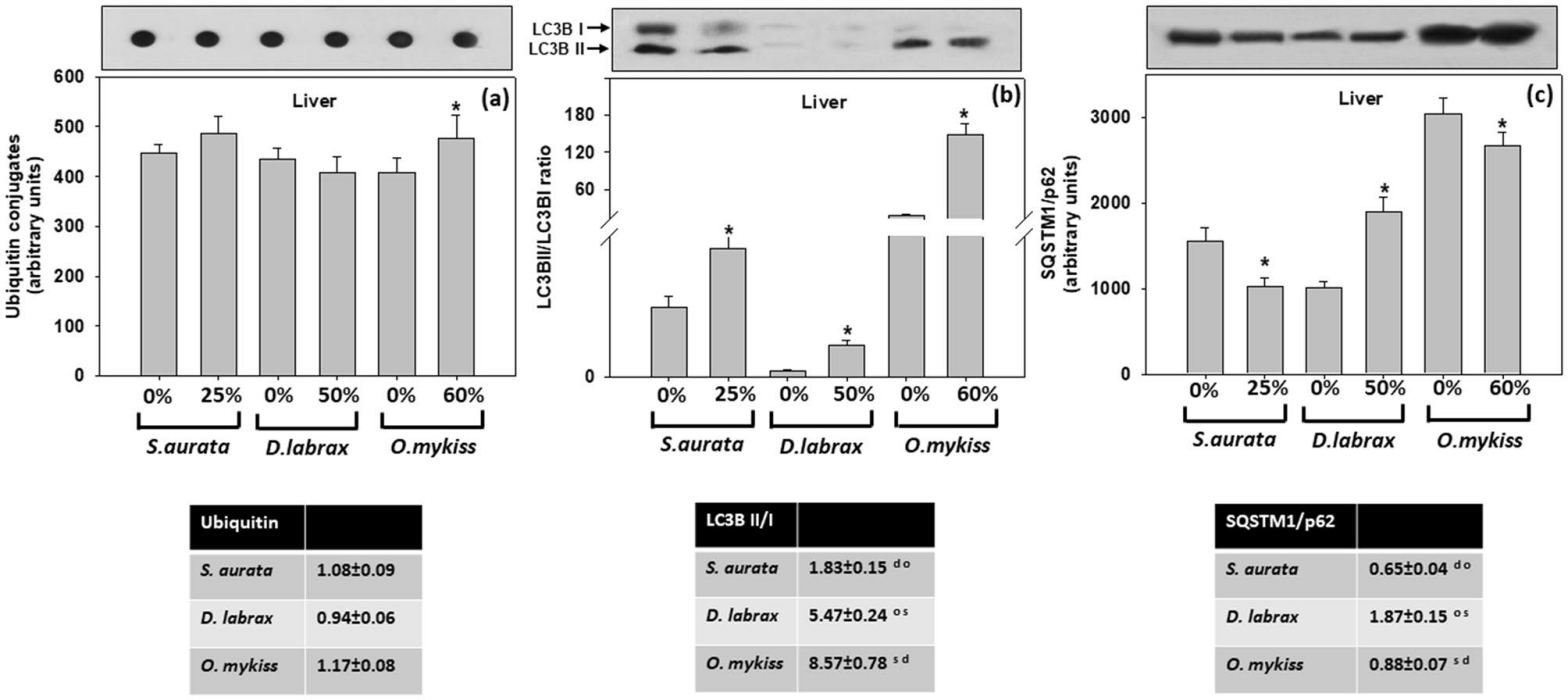
**(a**) Ubiquitin conjugates, (**b**) LC3BII/LC3BI ratio and (**c**) SQSTM1/p62 in the liver of gilthead seabream (*Sparus aurata*), European seabass (*Dicentrarchus labrax*) and rainbow trout (*Oncorhynchus mykiss*) under the 0–25%, 0–50% and 0–60% respectively inclusion of *T. molitor* (TM) meal. Representative immunoblots (Western blot for LC3BII/LC3BI and SQSTM1/p62; Dot blot for ubiquitin cojuagates) are shown and were quantified by laser scanning densitometry [Gel-Pro Analyzer 4.0 software (Media Cybernetics, Inc. www.mediacy.com)] and plotted [SigmaPlot 12.5 software (Systat Software Inc. www.systatsoftware.com)]. Values represent means ± SD; n = 5 preparations from different animals. Data was statistically analyzed [GraphPad Instat 3.10 (GraphPad Software, www.graphpad.com)]: *denotes significant differences (*p* < 0.05) compared with 0%. The accompanying tables exhibit fold-differences between TM diet inclusion and control (0%). s, d and o denote significant differences (*p* < 0.05) between *S. aurata, D. labrax and O. mykiss,* respectively.

Similar to the caspase-conjugated proteins, in the gilthead sea bream and European seabass, no significant differences were observed regarding ubiquitin levels in the liver of 0–25% and 0–50% insect (TM) meal inclusion treated fish, respectively (Fig. 4a). In the rainbow trout, the levels of ubiquitin-conjugated proteins were significantly increased (*p* < 0.05) in the liver of fish fed the 60% insect (TM) meal inclusion group, compared to the fish meal rich diet (Fig. 4a).

Concerning LC3BII/LC3BI ratio, in the liver of all three examined species, the insect meal replacement provoked significant increase (*p* < 0.05) in the levels of the above mentioned ratio (Fig. 4b).

In the gilthead sea bream, feeding with insect meal at 25% insect (TM) meal inclusion resulted in a significant reduction (*p* < 0.05) of SQSTM1/p62 in the liver relative to 0%, indicating autophagic activity. In the European seabass, the expression of SQSTM1/p62 was significantly increased (*p* < 0.05) in the 50% insect (TM) meal inclusion in the liver, while the opposite was observed in the rainbow trout liver at the 60% insect (TM) meal inclusion fish group (Fig. 4c).

Regarding interspecies differences (accompanying tables in Fig. 4), while the TM inclusion showed no differences in ubiquitination levels, statistically significant differences (expressed as x fold differences compared to control—0%) were found for SQSTM1/p62 levels and LC3II/I ratio between sea bream, the European seabass and rainbow trout.

## Discussion

Previous studies have demonstrated that insects can be used as an alternative protein source for fish feeds [e.g.^10,41–43^] and results on fish growth performances highly depend on insect meal type, inclusion rate, feed formulation, and, on the fish species and age^5^. The inclusion of TM larvae meal in gilthead sea bream diets was feasible up to 25%, while there were no negative effects on weight gain, crude protein and digestibility and marketable indexes in comparison to the control group (0% TM)^11^. Gasco et al.^9^ showed that in the European seabass of 5.23 g initial body weight a 50% insect (TM) meal inclusion resulted in a significant reduction in growth performance. Results for rainbow trout trial showed that a 60% TM inclusion resulted in a significant reduction in individual weight gain and feeding rate while other performance parameters were not affected by the TM substitution (unpublished data).

Information on the effect of dietary supplementation of fish meal on biochemical pathways is relatively scarce. However, differentiation in protein expression of apoptotic and cytoprotective pathways in response to dietary changes has been reported in few studies [e.g.,^17,44^] indicating nutritionally-induced stress. Partial fish meal supplementation with soybean meal in common dentex (*Dentex dentex*) exerted influence on several heat shock proteins (HSPs) and mitogen-activated protein kinases (MAPKs) expression in a tissue- and inclusion percentage- specific manner^17^. Furthermore, complementary mixture of plant protein as fish meal substitution led to modulation of hepatocyte apoptosis in hybrid grouper (*Epinephelus lanceolatus*♂ × *E. fuscoguttatus*♀) through down-regulation of apoptosis-related genes^44^. Modification in the European seabass cellular defense mechanisms concerning HSR (Heat Shock Response) has also been shown in response to fasting^45^. Nutrient intake has been previously characterized as a stressor that tends to directly affect HSPs expression and MAPK phosphorylation^46^. It is evident in this study that insect (TM) meal inclusion has an immediate effect on several pathways that influence apoptosis and autophagy. The observed stress effect may be attributed to the presence of chitin in the TM -based meal. Chitin is a naturally abundant long-chain polysaccharide found in the exoskeleton of a plethora of organisms such as the shell of crustaceans, the cuticle of insects and fungi and microorganisms cell wall^47^. Similar to our results, treatment of 3T3-L1 adipocytes with carboxymethyl chitin activated AMPK, which indicates the link between autophagy and the regulation of energy metabolism^48^. The intrinsic/Bcl-2-regulated/mitochondrial pathway is the main apoptosis signalling pathway^49^. Increase of pro-apoptotic to pro-survival proteins (Bax/Bcl-2) ratio determines the cellular resistance to several stressful stimuli, resulting in caspases activation which induces apoptosis^39,40^. Apoptosis can be activated by the inflammatory response initiated by the cellular damage due to oxidative stress^50^. Increased activity of antioxidant enzymes, indicating oxidative stress, was observed among others in the serum of Jian carp (*Cyprinus carpio* var. Jian) fed defatted *Hermetia illucens* meal^51^ and in the liver of pearl gentian grouper (*Epinephelus fuscoguttatus* × *E. lanceolatus*) fed TM meal^52^. In addition, taurine supplementation to primary liver cells of Atlantic salmon had been found to ameliorate the effects of CdCl_2_ on apoptosis by reducing caspace-3 activity while Bax and Bcl-2 levels were unaffected^53^.

By allowing the orderly cellular degradation and recycling of unnecessary or dysfunctional components, autophagy is a regulated cellular mechanism which can in general prevent apoptosis^54,55^. During autophagy, LC3-I is converted to LC3-II by an ubiquitin-like system that allows for LC3 to become associated with autophagosomes^56,57^. Moreover, SQSTM1/p62 interaction with ubiquitin, provides a scaffold for several signaling proteins and triggers degradation of proteins through the proteasome or lysosome^58^. Protein aggregates formed by SQSTM1/p62 can be degraded by the autophagosome^59,60^. The above seem to be consistent with our results concerning rainbow trout and European seabass, where insect (TM) meal inclusion provoked stress seems to induce hepatic autophagy which in turns inhibits apoptosis. On the other hand, gilthead seabream exhibits a different pattern of stress with increased levels of both autophagy and apoptosis. The latter is observed probably due to the fact that although autophagy serves as an anti-apoptotic process, when prolonged or in certain conditions (e.g., cells deprivation of oxygen and nutrients) cells will subsequently go through apoptosis^61,62^. It must be noted that insect meals appear to be limiting in several indispensable amino acids like methionine, lysine, histidine and tyrosine^3^. TM, specifically, has 39.2% less lysine, 34.4% less histidine and 48.4% less methionine than fish meal (Table 1, Feedtables^63^). In the present study, there was no supplementation of essential amino acids, namely methionine and lysine, in any of the substitution diets suggesting that these diets were deficient in those amino acids, and potentially others. Feed consumption of gilthead sea bream and European seabass was not increased to compensate for the amino acid limiting diets^9,11^, therefore an amino acid deprivation regime occurred. In cases of nutrient deprivation, when the availability of amino acids or glucose is not enough to sustain protein synthesis or other metabolic reactions, the rapid deactivation of the function of the target of rapamycin (TOR) induces autophagy to degrade and recycle cell components^64–66^. In the muscle of rainbow trout, a 14-day starvation led to an upregulation of autophagy related genes^67^ and in the muscle of Chinese perch, *Siniperca chuatsi,* a five day starvation led to upregulation of core autophagy related genes, increased formation of autophagosomes and autolysosomes and higher levels of LC3 protein^68^. In mammals, starvation leads to major activation of the ubiquitin–proteasome pathway for protein degradation, while the autophagic-lysosomal pathway plays a much smaller role, if activated at all^69^. In fish systems, the exact opposite applies. The lysosomal system is responsible for the 30–34% of protein degradation and the ubiquitin–proteasome system for the 4%^69,70^, which is in line with our data about the activation of the lysosomal pathway in all three species and the increase in ubiquitin-conjugated proteins only in rainbow trout, probably because of the high level of substitution. In the muscle of rainbow trout, methionine deficiency has been found to induce autophagy through both pathways^71^. After 6 weeks of feeding with a methionine deficient diet, in which the methionine content was 32% lower than the nutrient requirements, Belghit et al.^71^ observed an induction of the autophagic-lysosomal pathway and also upregulation of several proteasome related genes involved in the attachment of ubiquitin to substrates and the recognition of the ubiquitin conjugated proteins to the proteasome. Methionine is a sulphur amino acid which is essential for the growth of most animals not only because it is a constituent of body protein, but also because of its very important metabolic roles. Methionine is a major methyl-group donor and is involved in the production of cysteine, taurine and glutathione, among others^72^. Taurine, which is abundant in fish meal but not present in insect meals^73^, acts beneficially as an antioxidant agent by reducing lipid peroxidation levels, scavenging various radicals and modulating the production of reactive oxygen species^74^. Since autophagy is induced in cases of oxidative stress, it can be hypothesized that taurine protects proteins from oxidative damage leading to lower autophagic activity for protein degradation^75^. In meagre, *Argyrosomus regius*, 2% of taurine supplementation in plant-based diets decreased cathepsin activity in the liver, thus lowering the autophagy-lysosomal degradation of proteins^75^ and in pufferfish, *Takifugu obscurus,* exposed to low temperature stress, taurine supplementation led to a decrease in reactive oxygen species, an enhancement of the activity of antioxidant enzymes and a decrease of apoptosis through caspace-3 activity reduction^76^.

Proteomics is a well-established post-genomic tool which allows investigation in fish biology. Research in aquaculture uses this methodology to investigate issues for fish pathology, nutrition and physiology. Furthermore, there have been few attempts to determine the relationship between diet quality and nutrient utilization in fish liver proteome^26–28,32,77^. However, to the knowledge of the authors this is the first study that examines proteome analysis of liver tissue in two marine fish species and one freshwater, fed an insect meal diet. Differences regarding the liver proteome were found in each of the three- different fish species. Liver proteome comparisons in each species per two different treatments were made. Insect (TM) meal inclusion in fish diets has a more observable effect on the liver proteome of European seabass and gilthead sea bream*.* Nevertheless, in gilthead sea bream fewer proteins spots were altered in comparison to European seabass and rainbow trout after insect (TM) meal inclusion suggesting a possible relationship to the animal’s natural chitin-enriched diet^78,79^. Moreover, insect (TM) meal inclusion in European seabass and 60% in rainbow trout stimulates higher protein abundance changes and lower fish growth in comparison to the 0% insect (TM) meal inclusion. Composition and quality of dietary protein intake influence the changes in fish protein abundance. A lower non-structural protein expression level was observed by Martin et al.^28^ in rainbow trout (*O. mykiss*) fed a fishmeal and plant protein diet than a higher proportion of soy protein diet. Furthermore, previous studies in rainbow trout^28,29^ demonstrated changes in hepatic metabolism as a response to the dietary consumption of various plant proteins. Thus, the present protein profile analysis reflects a specific proteomic phenotype, which reinforces the knowledge regarding the biology of the fish.

## Conclusion

**Figure 5:**
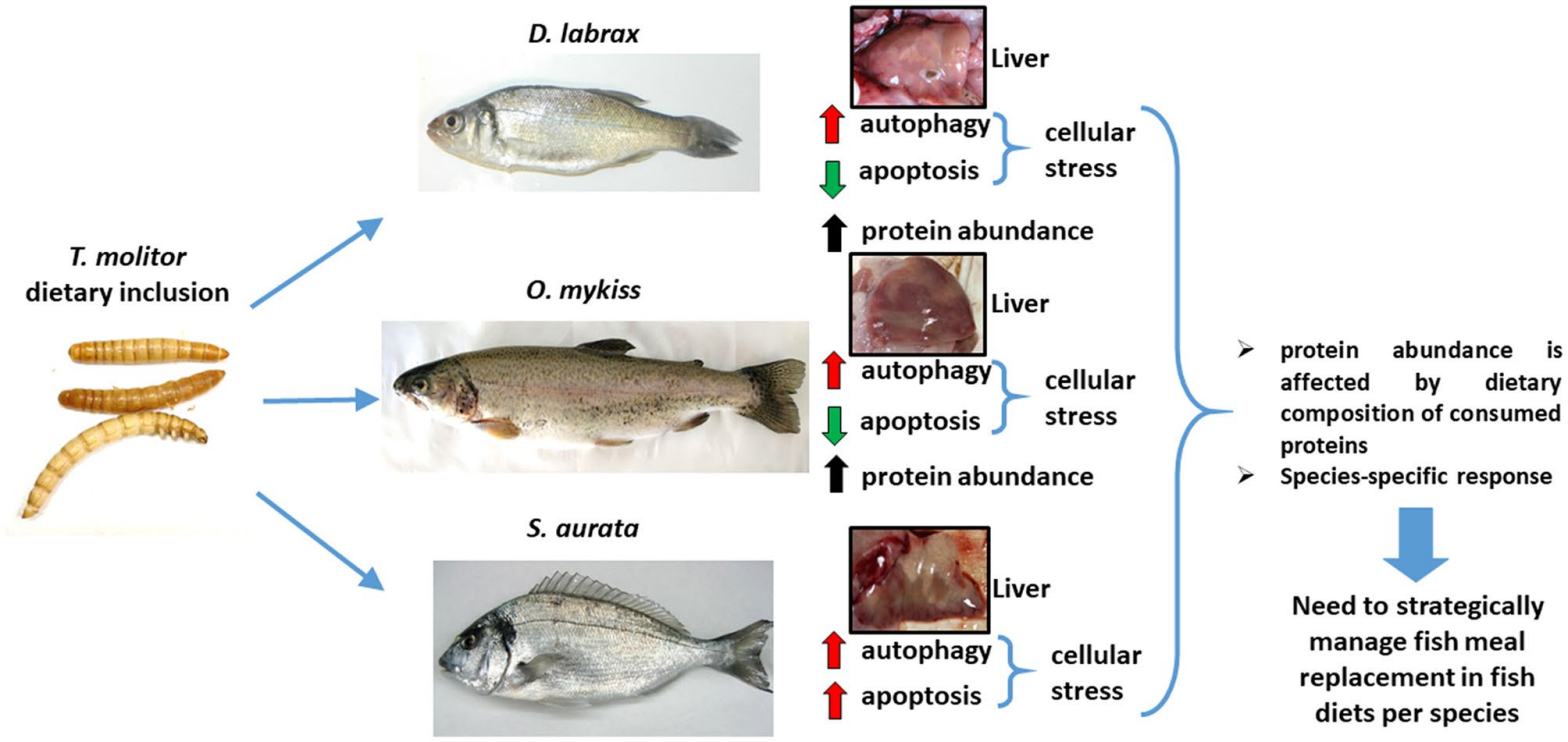
Graphical representation of the effect of *Tenebrio molitor* (TM) dietary inclusion on the hepatic apoptosis and autophagy, and the hepatic proteome profile of gilthead seabream (*Sparus aurata*), European seabass (*Dicentrarchus labrax*) the rainbow trout (*Oncorhynchus mykiss*). Photographs of *T. molitor* (TM) meal, fish species investigated, and liver form each species are copyright of our laboratory.

Alternative feed ingredients and protein sources for the aqua feed industry due to insufficient global supplies of FM^1,2^ is of great necessity in the aquaculture sector. Although insect larvae meals are considered as very promising alternative to provide valuable proteins for aqua feeds and have been in the spotlight of many researches, extensive study is needed for adequate management of fish resources. The present study has highlighted that although cellular stress was evident in the three teleost species following dietary TM inclusion, European seabass and rainbow trout were able to suppress apoptosis through induction of hepatic autophagy, while in gilthead seabream, both cellular procedures were activated. Along with the changes observed in apoptotic and autophagic pathway, which play a vital role to cell and organism homeostasis, changes in protein abundance are also affected by the dietary composition and quality of consumed proteins. This study also shows that insect meal, at least in terms of protein changes, is more suitable for species whose natural diet includes such ingredients. The results of this study indicate that the most desirable fish diet substitution differentially affects fish protein profile, implying that a species-specific tailor-made approach in diet manipulations should be considered in the future (Fig. 5). To our knowledge, this is the first study that showed that insect (TM) meal inclusion stimulates higher protein abundance changes and lower fish growth in European seabass and rainbow trout compared to gilthead sea bream. However, a species-specific response both in apoptosis and autophagy, as well as the abundance of liver proteins, indicates a need to strategically manage fish meal replacement in fish diets per species. The proteomics and cellular stress response approach described here could be used to further investigate biochemical pathways that are likely to be affected due to fish diet composition in aquaculture and to identify protein change most associated with fish species-specific physiology. This lays the basis for future research to explore in detail the identity of the altered proteins and allow for more in-depth interpretation of the metabolic and cellular responses, delivering further insights into fish nutrition etiology and providing sustainable feeding management practices.

## Supplementary Information

Supplementary Information.
