# Supplementary material for: Tenebrio molitor larvae meal inclusion affects hepatic proteome and apoptosis and/or autophagy of three farmed fish species
Source: Sci Rep. 2022 Jan 7;12:121. doi: 10.1038/s41598-021-03306-8 (PMC8742038; doi:10.1038/s41598-021-03306-8)

Tenebrio molitor larvae meal inclusion affects hepatic proteome and apoptosis and/or autophagy of three farmed fish species

Eleni Mente[1,*], Thomas Bousdras[2], Konstantinos Feidantsis[2], Nikolas Panteli[2], Maria Mastoraki[2], Konstantinos Ar. Kormas[1], Stavros Chatzifotis[3], Giovanni Piccolo[4], Laura Gasco[5], Francesco Gai[6], Samuel A.M. Martin,[7] Efthimia Antonopoulou[2]

[1]Department of Ichthyology and Aquatic Environment, School of Agricultural Sciences, University of Thessaly, 384 46 Volos, Greece.

[2]Laboratory of Animal Physiology, Department of Zoology, School of Biology, Aristotle University of Thessaloniki, 541 24 Thessaloniki, Greece

[3]Institute of Marine Biology, Biotechnology and Aquaculture, Hellenic Centre for Marine Research, Gournes Pediados P.O. Box 2214, 71003 Heraklion, Crete, Greece

[4] Department of Veterinary Medicine and Animal Production, University of Naples Federico II, Via F. Delpino 1, 80137 Naples, Italy

[5]Department of Agricultural, Forest and Food Sciences, University of Turin, Largo P. Braccini 2, 10095 Grugliasco, Italy

[6]Institute of Sciences of Food Production, National Research Council, Largo P. Braccini 2, 10095 Grugliasco, Italy

[7]School of Biological Sciences, University of Aberdeen, Tillydrone Avenue, Aberdeen, UK

* Corresponding author; Eleni Mente, email: emente@uth.gr

Original blots in supplementary information

Cropped blot in main paper

Ubiquitin conjugates

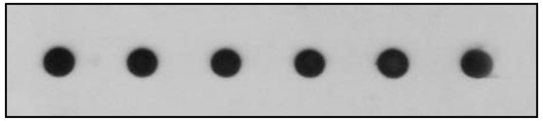

Caspases conjugates

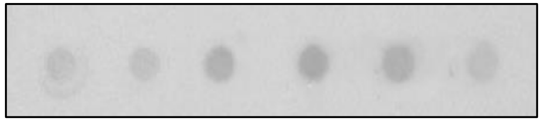

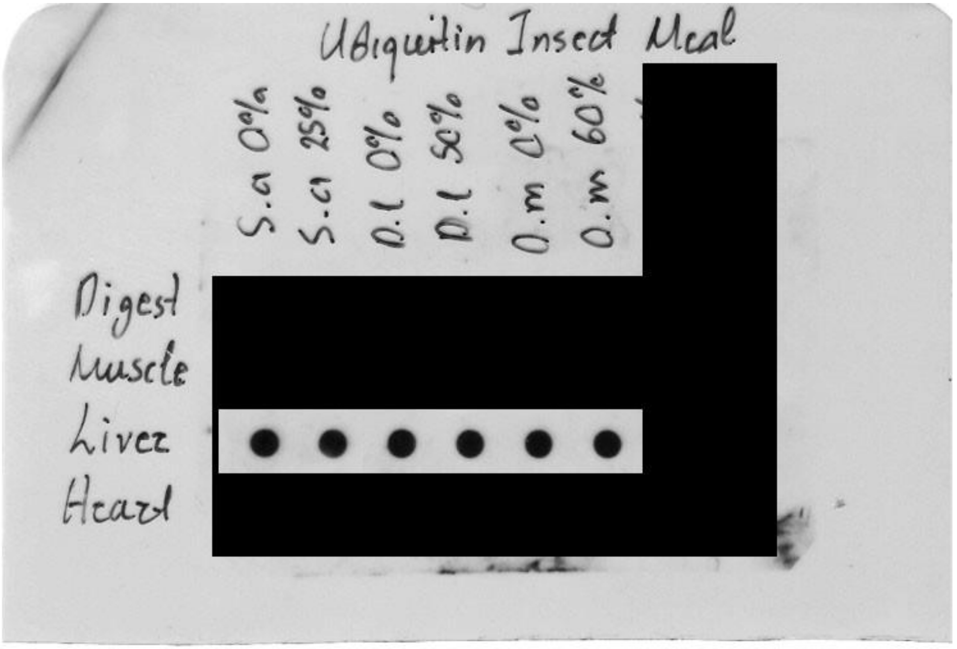

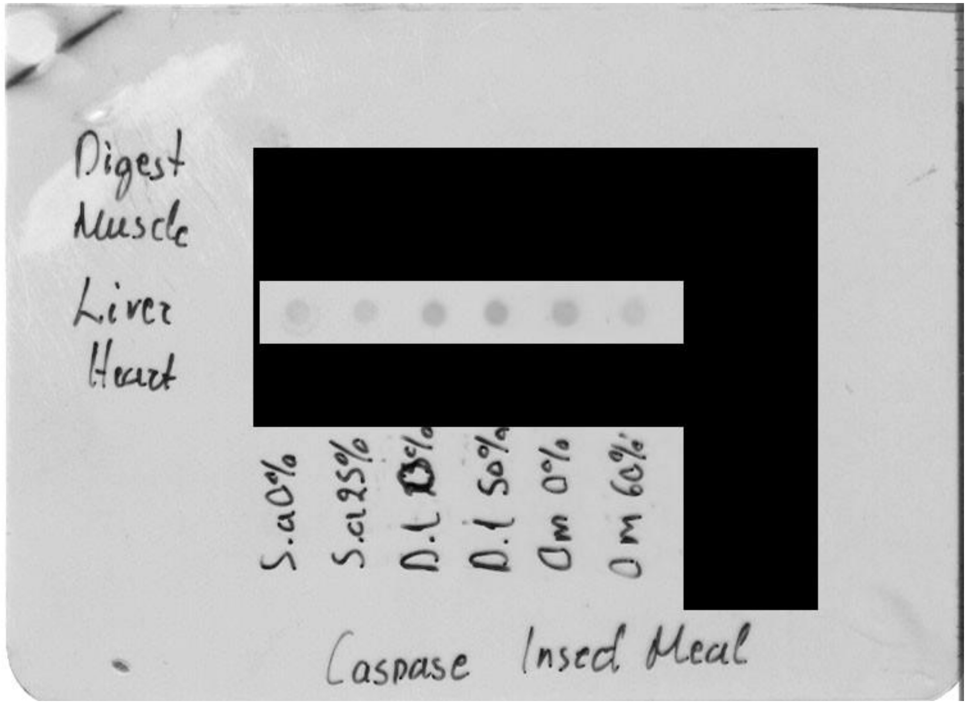

Original blots in supplementary information

Cropped blot in main paper

LC3B

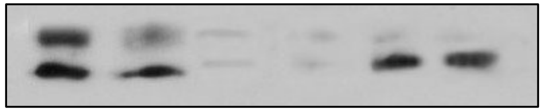

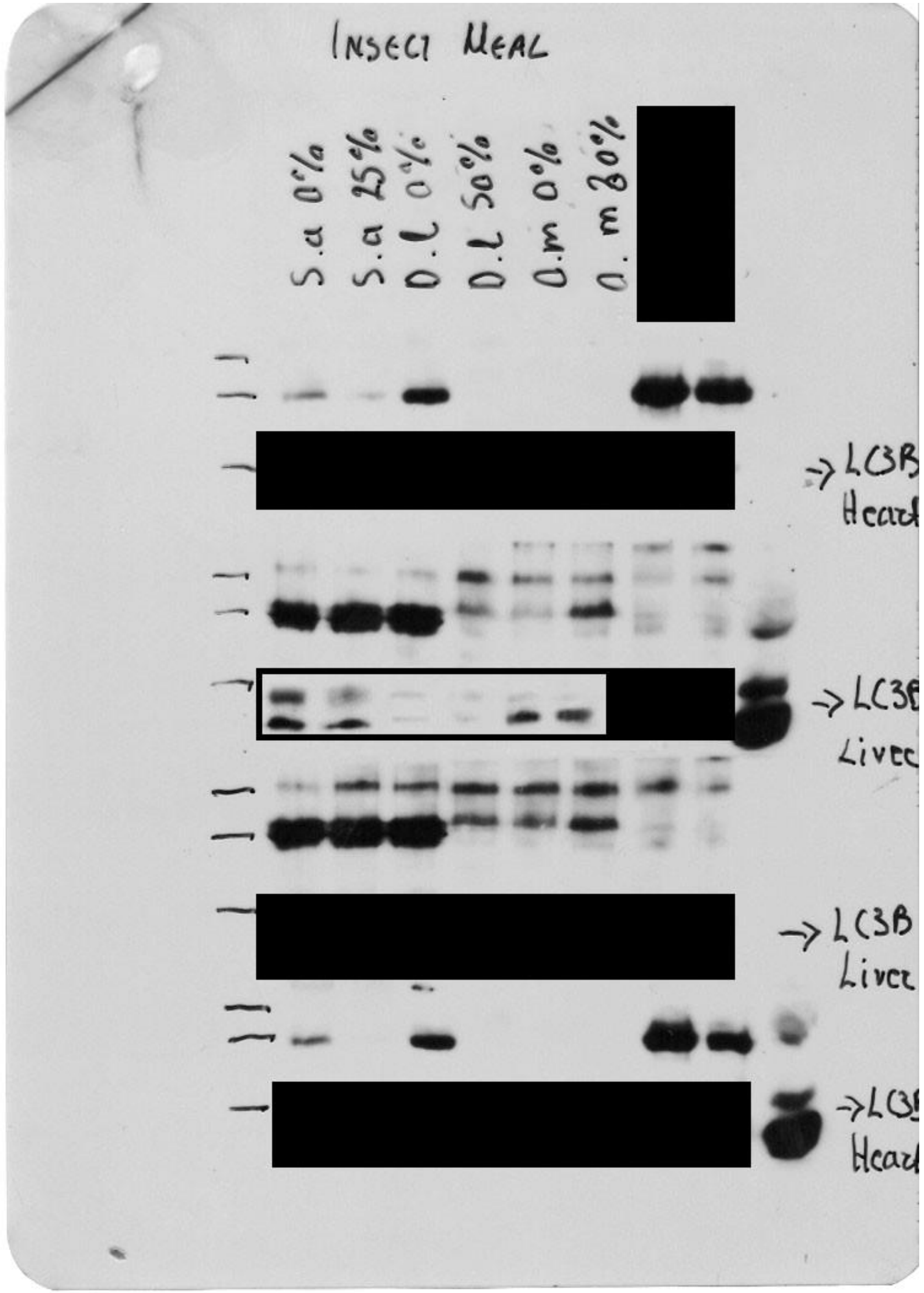

Cropped blot in main paper

SQSTM1/p62

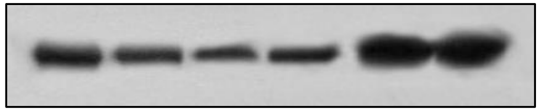

Original blots in supplementary information

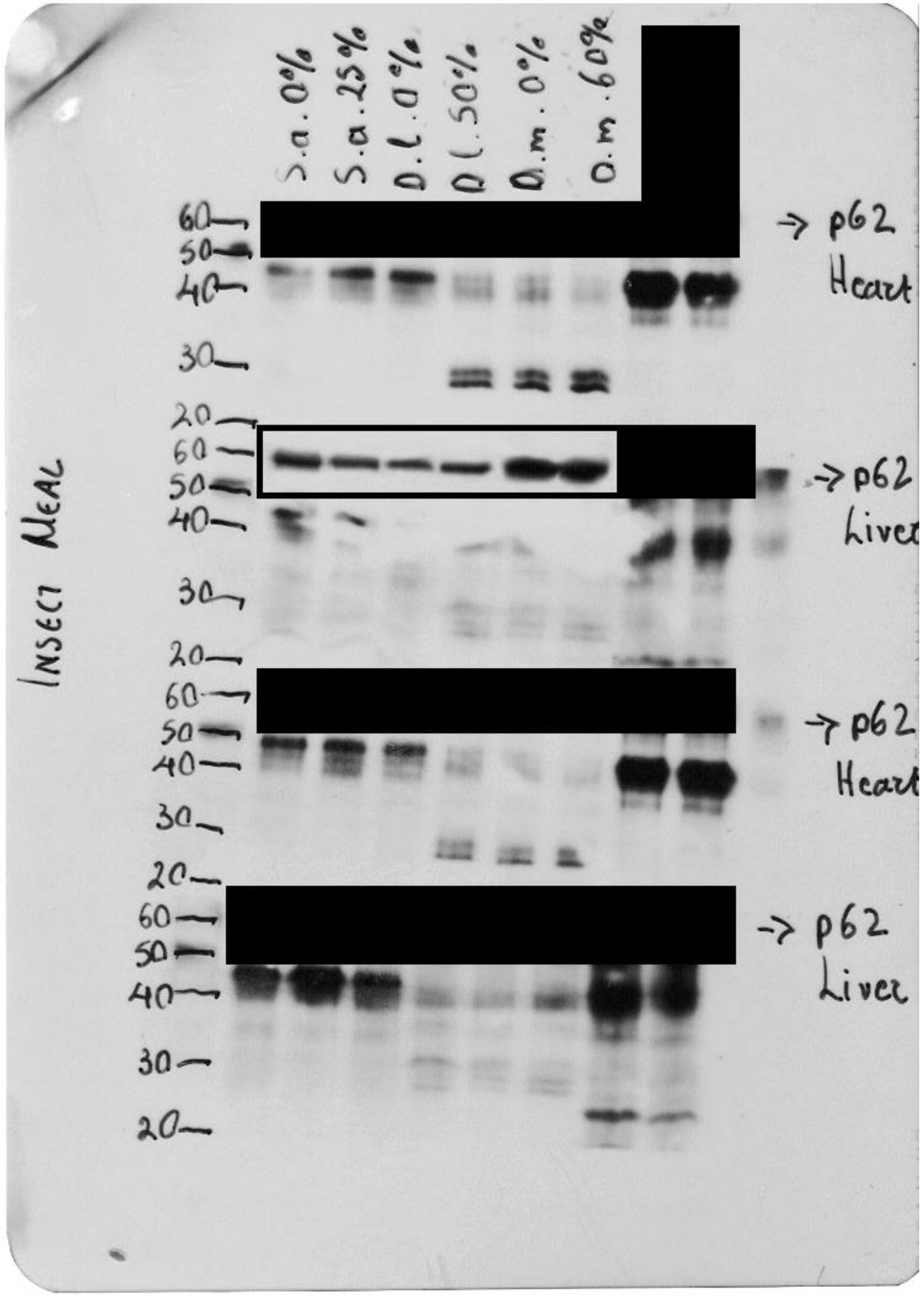

Cropped blot in main paper

Bax

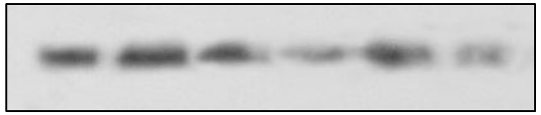

Original blots in supplementary information

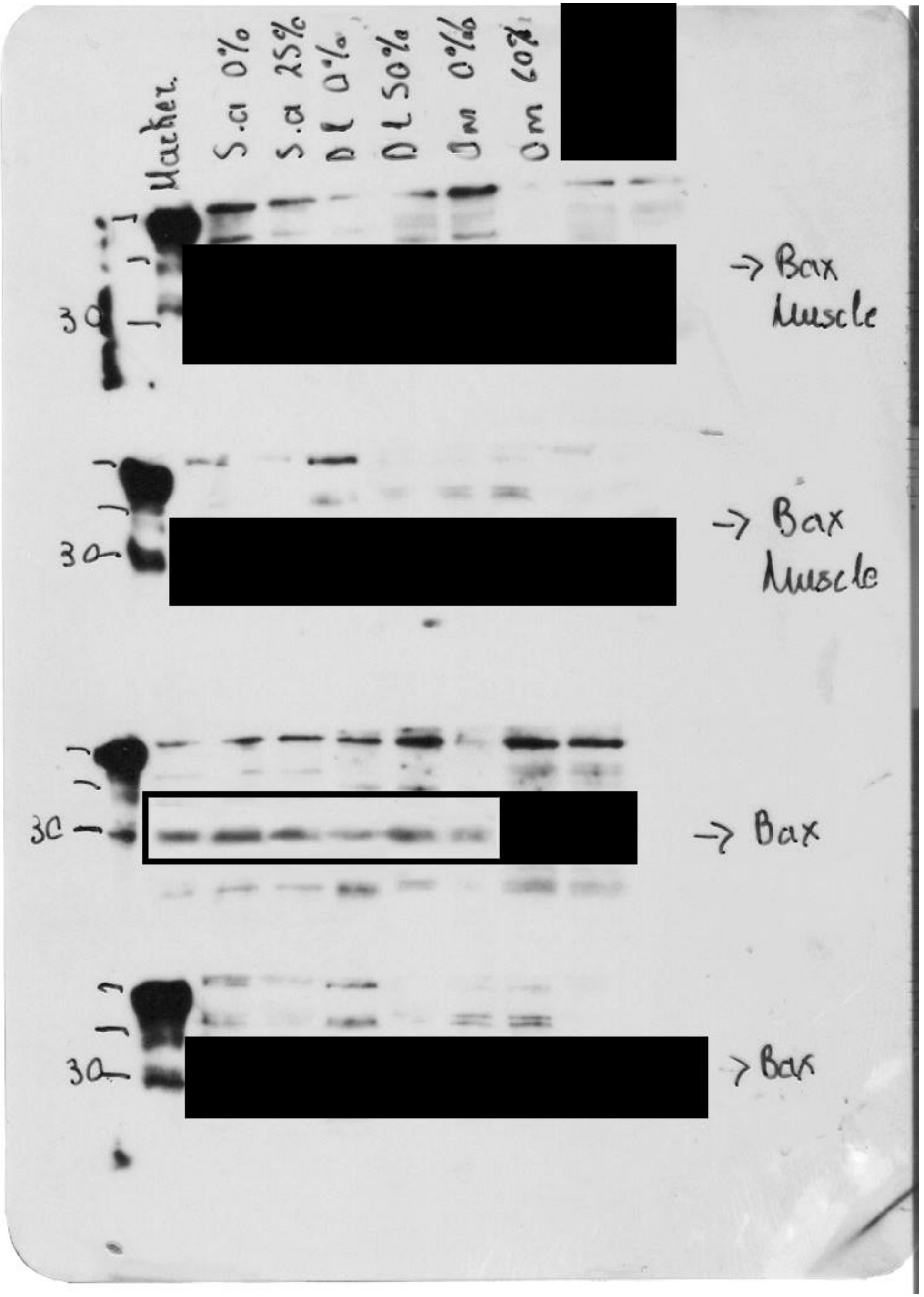

Original blots in supplementary information

Cropped blot in main paper

Bcl-2

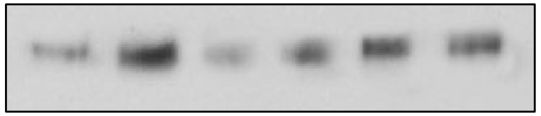

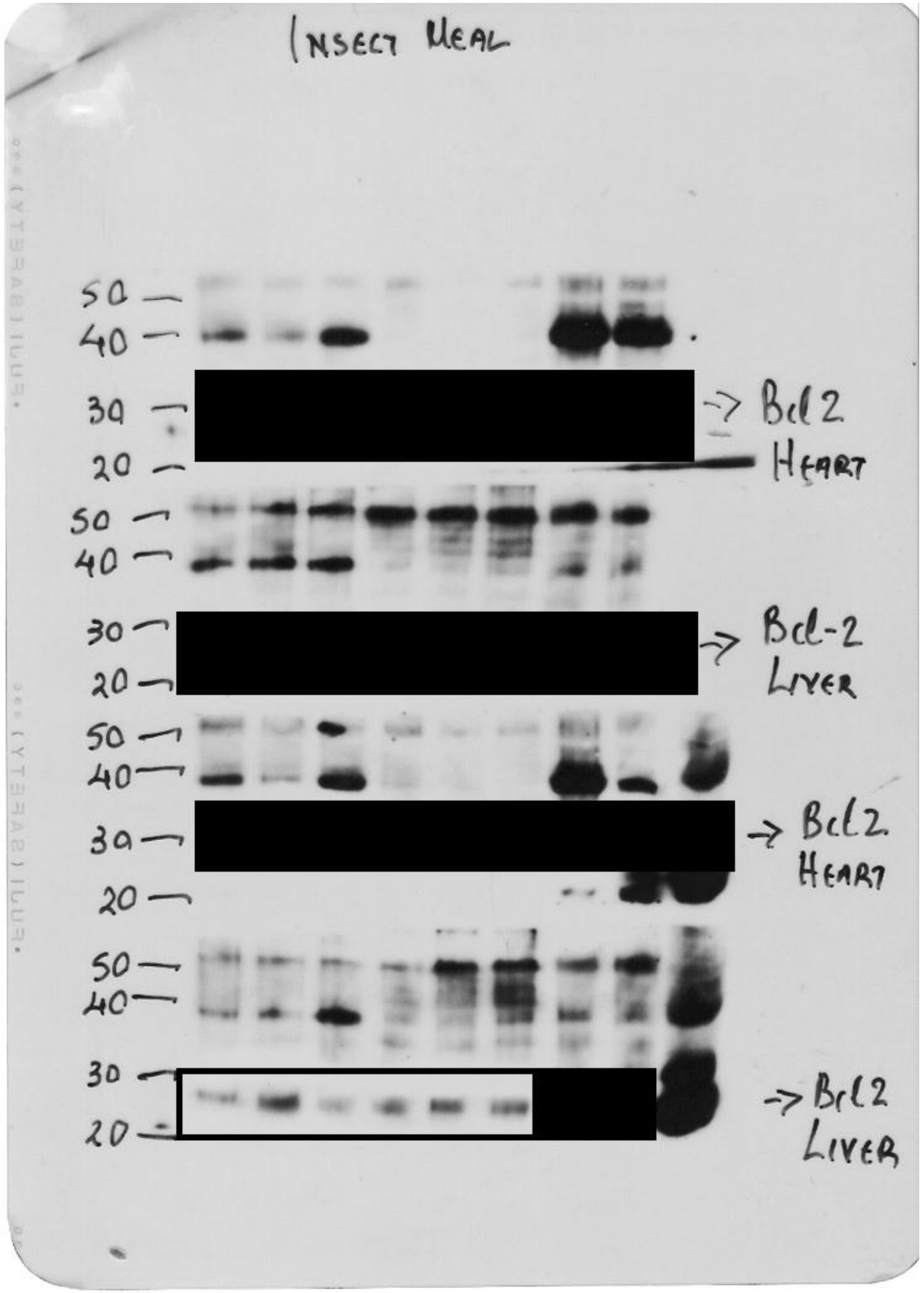

Supplement: Supplementary file 1 — Supplementary Information. [file 41598_2021_3306_MOESM1_ESM.pdf]
